# Crystal structure of 2-bromo-1,4-dihy­droxy-9,10-anthra­quinone

**DOI:** 10.1107/S1600536814020996

**Published:** 2014-09-27

**Authors:** Wataru Furukawa, Munenori Takehara, Yoshinori Inoue, Chitoshi Kitamura

**Affiliations:** a Department of Materials Science, School of Engineering, The University of Shiga Prefecture, 2500 Hassaka-cho, Hikone, Shiga 522-8533, Japan

**Keywords:** crystal structure, 1,4-dihy­droxy-9,10-anthraquione, hydrogen bonds

## Abstract

In an attempt to brominate 1,4-diprop­oxy-9,10-anthra­quinone, a mixture of products, including the title compound, C_14_H_7_BrO_4_, was obtained. The mol­ecule is essentially planar (r.m.s. deviation = 0.029 Å) and two intra­molecular O—H⋯O hydrogen bonds occur. In the crystal, the mol­ecules are linked by weak C—H⋯O hydrogen bonds, Br⋯O contacts [3.240 (5) Å], and π–π stacking inter­actions [shortest centroid–centroid separation = 3.562 (4) Å], generating a three-dimensional network.

## Related literature   

For the original synthesis of the title compound, see: Peters & Tenny (1977 ▶). For related crystal structures of 1,4-dihy­droxy-9,10-anthra­quinone derivatives, see: Nigam & Deppisch (1980 ▶); Hall *et al.* (1988 ▶). For 1,4-diprop­oxy-9,10-anthra­quinone, see: Kitamura *et al.* (2004 ▶).
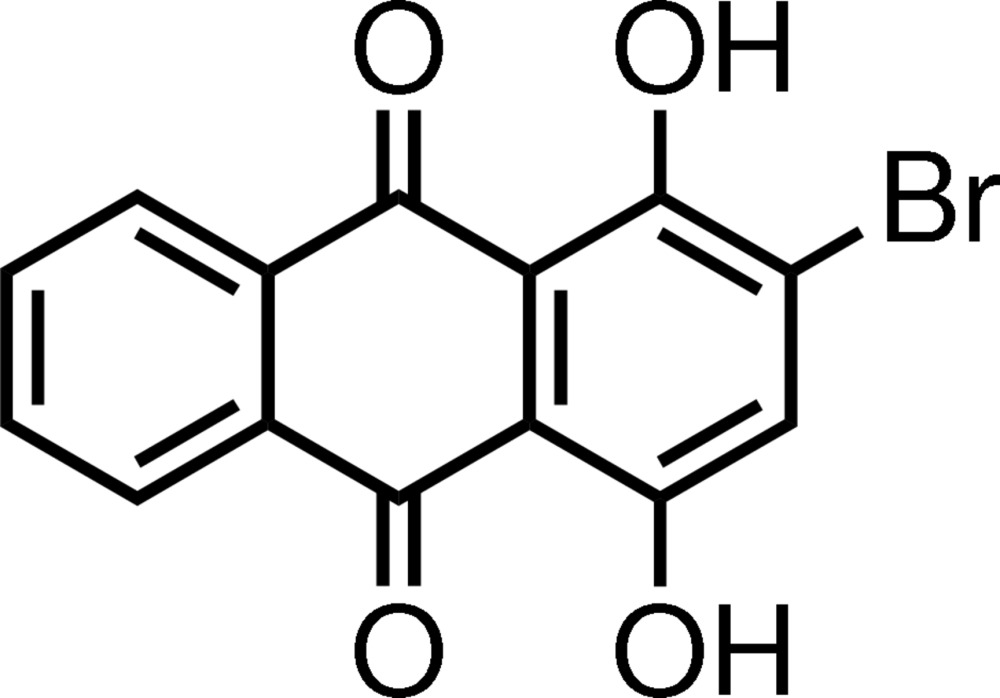


## Experimental   

### Crystal data  

C_14_H_7_BrO_4_
*M* *_r_* = 319.11
Orthorhombic, 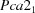

*a* = 18.977 (3) Å
*b* = 3.7811 (4) Å
*c* = 15.5047 (18) Å
*V* = 1112.5 (2) Å^3^
*Z* = 4
Mo *K*α radiation
μ = 3.70 mm^−1^
*T* = 200 K
0.5 × 0.4 × 0.05 mm

### Data collection  

Rigaku R-AXIS RAPID diffractometer
Absorption correction: numerical (*NUMABS*; Higashi, 1999 ▶) *T*_min_ = 0.322, *T*_max_ = 0.912
15045 measured reflections
2476 independent reflections
2103 reflections with *I* > 2σ(*I*)
*R*_int_ = 0.091

### Refinement  

*R*[*F* ^2^ > 2σ(*F* ^2^)] = 0.057
*wR*(*F* ^2^) = 0.099
*S* = 1.11
2476 reflections
179 parameters
1 restraint
H atoms treated by a mixture of independent and constrained refinement
Δρ_max_ = 0.85 e Å^−3^
Δρ_min_ = −1.36 e Å^−3^
Absolute structure: Flack *x* determined using 846 quotients [(*I* ^+^)−(*I* ^−^)]/[(*I* ^+^)+(*I* ^−^)] (Parsons & Flack, 2004 ▶)
Absolute structure parameter: 0.000 (11)

### 

Data collection: *PROCESS-AUTO* (Rigaku, 1998 ▶); cell refinement: *PROCESS-AUTO*; data reduction: *PROCESS-AUTO*; program(s) used to solve structure: *SIR2004* (Burla *et al.*, 2005 ▶); program(s) used to refine structure: *SHELXL2013* (Sheldrick, 2008 ▶); molecular graphics: *Mercury* (Macrae *et al.*, 2008 ▶); software used to prepare material for publication: *WinGX* (Farrugia, 2012 ▶).

## Supplementary Material

Crystal structure: contains datablock(s) I, global. DOI: 10.1107/S1600536814020996/hb7282sup1.cif

Structure factors: contains datablock(s) I. DOI: 10.1107/S1600536814020996/hb7282Isup2.hkl

Supporting information file. DOI: 10.1107/S1600536814020996/hb7282Isup3.cml

**Fig. 1.**
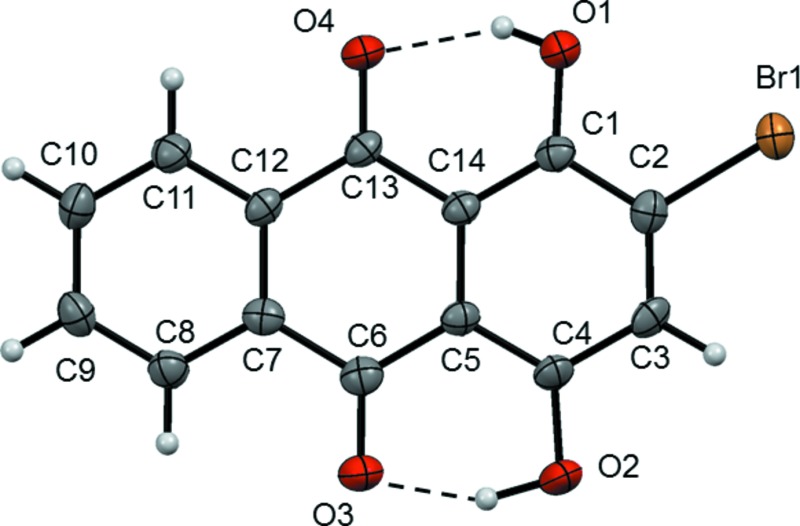
The molecular structure of (I), showing 50% probability displacement ellipsoids. The intramolecular hydrogen bonds are drawn by dashed lines.

. DOI: 10.1107/S1600536814020996/hb7282fig1.tif
The mol­ecular structure of (I), showing 50% probability displacement ellipsoids. The intra­molecular hydrogen bonds are drawn by dashed lines.

**Fig. 2.**
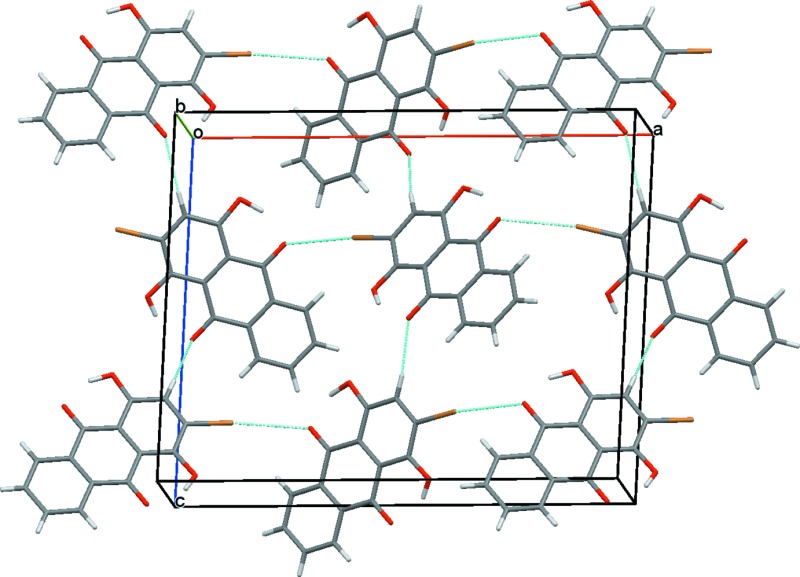
The crystal packing of (I), showing short contacts of selected C–H···O and Br···O interactions by blue lines.

. DOI: 10.1107/S1600536814020996/hb7282fig2.tif
The crystal packing of (I), showing short contacts of selected C–H⋯O and Br⋯O inter­actions by blue lines.

CCDC reference: 1025364

Additional supporting information:  crystallographic information; 3D view; checkCIF report

## Figures and Tables

**Table 1:** Hydrogen-bond geometry (Å, °)

*D*—H⋯*A*	*D*—H	H⋯*A*	*D*⋯*A*	*D*—H⋯*A*
O1—H1⋯O4	0.80 (9)	1.82 (9)	2.536 (8)	148 (9)
O2—H2⋯O3	0.99 (10)	1.65 (10)	2.568 (9)	152 (8)
C3—H3⋯O4^i^	0.95	2.46	3.396 (9)	169
C9—H9⋯O1^ii^	0.95	2.59	3.308 (10)	133
C9—H9⋯O2^iii^	0.95	2.69	3.394 (10)	132

Symmetry codes: (i) 
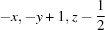
; (ii) 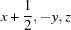; (iii) 
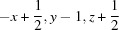
.

## supplementary crystallographic information

### S1. Comment

Anthraquinone and its derivatives are important dyestuff molecules. In this work, we attempted to brominate 1,4-dipropoxy-9,10-anthraquinone (Kitamura *et al.*, 2004) with elementary bromine in acetic acid to obtain 2-bromo-1,4-dipropoxy-9,10-anthraquinone. As a result, the reaction afforded a complex mixture of products containing 2-bromo-1,4-dihydroxy-9,10-anthraquinone, C_14_H_7_BrO_4_ (I). Synthesis of the title compound, (I), was already reported by Peters & Tenny (1977) using a different method. However, the X-ray structure of (I) was not reported so far. We report here the crystal structure of the title compound, (I).

The title compound crystallizes in the orthorhombic space group *Pca*2_1_ with a Flack parameter of 0.000 (11). The molecular structure of (I) is shown in Figure 1. The molecule is nearly planar with the maximum deviation of 0.053 (7) Å for O2. The bond length of C6—O3 and C13—O4 is 1.246 (9) Å and 1.238 (9) Å, respectively. The length of the single C—O bond of C1—O1 and C4—O2 is 1.340 (9) Å and 1.348 (10) Å, respectively. There are two intramolecular hydrogen bonds, O1—H1···O4 and O2—H2···O3. The distance of O1—O4 and O2—O3 is 2.536 (8) Å and 2.568 (9) Å, respectively. These values are in good agreement with those observed for 1,4-dihydroxy-9,10-anthraquinone (Nigam & Deppisch, 1980) and 2,3-dichloro-1,4-dihydroxy-9,10-anthraquinone (Hall *et al.*, 1988).

As shown in Figure 2, in the crystal, molecules are linked by C—H···O hydrogen bonds (Table 1) and Br···O contacts [Br1···O3^i^ = 3.240 (5) Å; symmetry code: (i) *x* - 1/2, -*y* + 1, *z*], whose value is shorter than the sum of van der Waals radii of bromine and oxygen atoms. The molecules are π-stacked along the *a* axis with an interplanar distance of 3.450 Å.

### S2. Experimental

A mixture of 1,4-dipropoxy-9,10-anthraquinone (653 mg, 2.01 mmol), iron powder (50 mg, 0.89 mmol), bromine (0.40 g, 2.5 mmol) in acetic acid (20 ml) was stirred at 80 °C under air. The reaction was quenched with an aqueous solution of Na_2_SO_3_. Then the reaction products were precipitated. After filtration, the residue was subjected to column chromatography on silica gel using CH_2_Cl_2_-hexane as the eluent to afford the title compound (18 mg, 2.8% yield) as a red solid. Red platelets were obtained by slow evaporation of a CH_2_Cl_2_ solution.

### S3. Refinement

All the aromatic H atoms were positioned geometrically and refined using a riding model with C—H = 0.95 Å and with *U*_iso_(H) = 1.2*U*_eq_(C). The H atoms of the OH groups were located in a difference Fourier map and freely refined [O1—H1 = 0.80 (9) Å and O2—H2 = 0.99 (10) Å].

### Crystal data

**Table:**

C_14_H_7_BrO_4_	*D*_x_ = 1.905 Mg m^−^^3^
*M**_r_* = 319.11	Melting point: 485 K
Orthorhombic, *P**c**a*2_1_	Mo *K*α radiation, λ = 0.71073 Å
Hall symbol: P 2c -2ac	Cell parameters from 12445 reflections
*a* = 18.977 (3) Å	θ = 3.4–27.5°
*b* = 3.7811 (4) Å	µ = 3.70 mm^−^^1^
*c* = 15.5047 (18) Å	*T* = 200 K
*V* = 1112.5 (2) Å^3^	Platelet, red
*Z* = 4	0.5 × 0.4 × 0.05 mm
*F*(000) = 632	

### Data collection

**Table:**

Rigaku R-AXIS RAPID diffractometer	2476 independent reflections
Radiation source: fine-focus sealed x-ray tube	2103 reflections with *I* > 2σ(*I*)
Graphite monochromator	*R*_int_ = 0.091
Detector resolution: 10 pixels mm^-1^	θ_max_ = 27.5°, θ_min_ = 3.4°
ω scans	*h* = −24→24
Absorption correction: numerical (*NUMABS*; Higashi, 1999)	*k* = −4→4
*T*_min_ = 0.322, *T*_max_ = 0.912	*l* = −20→20
15045 measured reflections	

### Refinement

**Table:**

Refinement on *F*^2^	Hydrogen site location: mixed
Least-squares matrix: full	H atoms treated by a mixture of independent and constrained refinement
*R*[*F*^2^ > 2σ(*F*^2^)] = 0.057	*w* = 1/[σ^2^(*F*_o_^2^) + (0.0171*P*)^2^ + 1.2737*P*] where *P* = (*F*_o_^2^ + 2*F*_c_^2^)/3
*wR*(*F*^2^) = 0.099	(Δ/σ)_max_ = 0.001
*S* = 1.11	Δρ_max_ = 0.85 e Å^−^^3^
2476 reflections	Δρ_min_ = −1.36 e Å^−^^3^
179 parameters	Extinction correction: *SHELXL*, Fc^*^=kFc[1+0.001xFc^2^λ^3^/sin(2θ)]^-1/4^
1 restraint	Extinction coefficient: 0.017 (2)
0 constraints	Absolute structure: Flack *x* determined using 846 quotients [(*I*^+^)-(*I*^-^)]/[(*I*^+^)+(*I*^-^)] (Parsons & Flack, 2004)
Primary atom site location: structure-invariant direct methods	Absolute structure parameter: 0.000 (11)

### Special details

**Table:**

Geometry. All e.s.d.'s (except the e.s.d. in the dihedral angle between two l.s. planes) are estimated using the full covariance matrix. The cell e.s.d.'s are taken into account individually in the estimation of e.s.d.'s in distances, angles and torsion angles; correlations between e.s.d.'s in cell parameters are only used when they are defined by crystal symmetry. An approximate (isotropic) treatment of cell e.s.d.'s is used for estimating e.s.d.'s involving l.s. planes.

### Fractional atomic coordinates and isotropic or equivalent isotropic displacement parameters (Å^2^)

**Table:**

	*x*	*y*	*z*	*U*_iso_*/*U*_eq_	
Br1	−0.12607 (3)	0.68820 (18)	0.29479 (9)	0.0301 (2)	
O1	−0.0661 (3)	0.3974 (18)	0.4573 (4)	0.0354 (15)	
O2	0.1215 (3)	0.3132 (19)	0.1883 (4)	0.0381 (15)	
O3	0.2118 (2)	0.0436 (14)	0.2927 (4)	0.0350 (11)	
O4	0.0247 (3)	0.1085 (17)	0.5543 (3)	0.0361 (15)	
C1	−0.0179 (4)	0.373 (2)	0.3944 (5)	0.0242 (16)	
C2	−0.0352 (4)	0.4934 (18)	0.3106 (5)	0.0258 (17)	
C3	0.0105 (4)	0.476 (2)	0.2437 (5)	0.0259 (17)	
H3	−0.0024	0.5634	0.1885	0.031*	
C4	0.0786 (5)	0.325 (2)	0.2576 (5)	0.0262 (18)	
C5	0.0979 (4)	0.209 (2)	0.3392 (5)	0.0222 (17)	
C6	0.1685 (4)	0.061 (2)	0.3529 (5)	0.0271 (17)	
C7	0.1876 (4)	−0.062 (2)	0.4400 (5)	0.0236 (15)	
C8	0.2550 (4)	−0.196 (2)	0.4556 (5)	0.0280 (17)	
H8	0.2885	−0.2062	0.4101	0.034*	
C9	0.2731 (4)	−0.312 (2)	0.5369 (6)	0.0304 (18)	
H9	0.319	−0.4027	0.5471	0.037*	
C10	0.2248 (5)	−0.299 (2)	0.6038 (6)	0.0310 (19)	
H10	0.2378	−0.3771	0.6598	0.037*	
C11	0.1572 (4)	−0.171 (2)	0.5885 (5)	0.0282 (17)	
H11	0.1239	−0.1641	0.6342	0.034*	
C12	0.1385 (4)	−0.054 (2)	0.5078 (5)	0.0228 (16)	
C13	0.0675 (4)	0.0989 (19)	0.4941 (5)	0.0234 (15)	
C14	0.0487 (4)	0.2263 (19)	0.4077 (5)	0.0226 (15)	
H1	−0.051 (5)	0.30 (2)	0.499 (6)	0.034*	
H2	0.164 (5)	0.20 (2)	0.212 (6)	0.034*	

### Atomic displacement parameters (Å^2^)

**Table:**

	*U*^11^	*U*^22^	*U*^33^	*U*^12^	*U*^13^	*U*^23^
Br1	0.0287 (4)	0.0284 (4)	0.0332 (4)	0.0024 (3)	−0.0052 (4)	0.0010 (8)
O1	0.030 (3)	0.052 (4)	0.024 (3)	0.008 (3)	0.002 (2)	0.003 (3)
O2	0.033 (3)	0.061 (4)	0.021 (3)	0.002 (3)	0.006 (2)	0.007 (3)
O3	0.031 (3)	0.048 (3)	0.025 (2)	0.003 (2)	0.006 (3)	0.000 (5)
O4	0.032 (4)	0.055 (5)	0.022 (3)	0.007 (3)	0.006 (2)	0.003 (3)
C1	0.025 (4)	0.026 (5)	0.021 (3)	−0.004 (3)	0.000 (3)	−0.003 (4)
C2	0.029 (4)	0.021 (4)	0.028 (5)	−0.002 (2)	−0.004 (3)	0.001 (4)
C3	0.038 (5)	0.017 (4)	0.023 (4)	−0.002 (3)	0.001 (3)	0.007 (3)
C4	0.027 (5)	0.028 (5)	0.023 (4)	−0.003 (3)	0.003 (3)	0.007 (4)
C5	0.025 (4)	0.018 (4)	0.023 (4)	−0.002 (3)	0.003 (3)	0.002 (4)
C6	0.030 (4)	0.025 (4)	0.026 (4)	−0.002 (3)	0.001 (3)	−0.003 (4)
C7	0.025 (4)	0.021 (4)	0.025 (4)	−0.003 (3)	0.002 (3)	−0.005 (3)
C8	0.025 (4)	0.028 (4)	0.031 (4)	0.000 (3)	0.003 (3)	0.004 (4)
C9	0.022 (4)	0.031 (4)	0.039 (5)	0.001 (3)	−0.003 (4)	0.002 (5)
C10	0.034 (5)	0.030 (5)	0.029 (4)	−0.001 (3)	−0.005 (4)	0.006 (5)
C11	0.029 (4)	0.031 (4)	0.024 (4)	0.000 (3)	0.000 (3)	−0.002 (4)
C12	0.030 (4)	0.017 (4)	0.022 (4)	−0.002 (3)	0.001 (3)	0.002 (3)
C13	0.030 (4)	0.022 (4)	0.019 (3)	−0.002 (3)	0.000 (3)	−0.001 (3)
C14	0.026 (4)	0.021 (4)	0.020 (3)	−0.001 (3)	0.001 (3)	−0.002 (3)

### Geometric parameters (Å, º)

**Table:**

Br1—C2	1.890 (7)	C5—C6	1.467 (12)
O1—C1	1.340 (9)	C6—C7	1.474 (11)
O1—H1	0.80 (9)	C7—C8	1.396 (11)
O2—C4	1.348 (10)	C7—C12	1.405 (10)
O2—H2	0.99 (10)	C8—C9	1.379 (12)
O3—C6	1.246 (9)	C8—H8	0.95
O4—C13	1.238 (9)	C9—C10	1.384 (13)
C1—C14	1.397 (10)	C9—H9	0.95
C1—C2	1.414 (10)	C10—C11	1.391 (12)
C2—C3	1.354 (10)	C10—H10	0.95
C3—C4	1.431 (12)	C11—C12	1.375 (11)
C3—H3	0.95	C11—H11	0.95
C4—C5	1.387 (10)	C12—C13	1.481 (10)
C5—C14	1.416 (10)	C13—C14	1.467 (10)
			
C1—O1—H1	108 (7)	C12—C7—C6	120.9 (7)
C4—O2—H2	102 (5)	C9—C8—C7	120.0 (7)
O1—C1—C14	122.5 (7)	C9—C8—H8	120
O1—C1—C2	119.2 (7)	C7—C8—H8	120
C14—C1—C2	118.3 (7)	C8—C9—C10	120.6 (8)
C3—C2—C1	122.6 (7)	C8—C9—H9	119.7
C3—C2—Br1	120.2 (6)	C10—C9—H9	119.7
C1—C2—Br1	117.1 (5)	C9—C10—C11	119.7 (8)
C2—C3—C4	118.8 (7)	C9—C10—H10	120.2
C2—C3—H3	120.6	C11—C10—H10	120.2
C4—C3—H3	120.6	C12—C11—C10	120.4 (7)
O2—C4—C5	123.9 (8)	C12—C11—H11	119.8
O2—C4—C3	116.0 (7)	C10—C11—H11	119.8
C5—C4—C3	120.2 (7)	C11—C12—C7	120.1 (7)
C4—C5—C14	119.7 (7)	C11—C12—C13	119.4 (7)
C4—C5—C6	119.6 (7)	C7—C12—C13	120.4 (7)
C14—C5—C6	120.7 (7)	O4—C13—C14	121.3 (7)
O3—C6—C5	120.9 (8)	O4—C13—C12	120.0 (7)
O3—C6—C7	120.5 (7)	C14—C13—C12	118.6 (6)
C5—C6—C7	118.6 (7)	C1—C14—C5	120.3 (7)
C8—C7—C12	119.2 (7)	C1—C14—C13	119.1 (7)
C8—C7—C6	119.9 (7)	C5—C14—C13	120.6 (7)
			
O1—C1—C2—C3	−179.7 (7)	C9—C10—C11—C12	−0.7 (14)
C14—C1—C2—C3	−1.1 (11)	C10—C11—C12—C7	−0.1 (13)
O1—C1—C2—Br1	1.0 (9)	C10—C11—C12—C13	−176.8 (7)
C14—C1—C2—Br1	179.6 (5)	C8—C7—C12—C11	1.0 (12)
C1—C2—C3—C4	1.2 (12)	C6—C7—C12—C11	179.8 (8)
Br1—C2—C3—C4	−179.5 (6)	C8—C7—C12—C13	177.6 (7)
C2—C3—C4—O2	179.5 (7)	C6—C7—C12—C13	−3.6 (11)
C2—C3—C4—C5	−2.2 (13)	C11—C12—C13—O4	−2.7 (12)
O2—C4—C5—C14	−178.8 (7)	C7—C12—C13—O4	−179.3 (8)
C3—C4—C5—C14	3.0 (14)	C11—C12—C13—C14	179.3 (7)
O2—C4—C5—C6	−0.7 (15)	C7—C12—C13—C14	2.6 (11)
C3—C4—C5—C6	−178.8 (7)	O1—C1—C14—C5	−179.6 (7)
C4—C5—C6—O3	1.6 (13)	C2—C1—C14—C5	1.9 (11)
C14—C5—C6—O3	179.6 (7)	O1—C1—C14—C13	−1.2 (11)
C4—C5—C6—C7	−179.6 (8)	C2—C1—C14—C13	−179.8 (6)
C14—C5—C6—C7	−1.5 (11)	C4—C5—C14—C1	−2.9 (12)
O3—C6—C7—C8	0.7 (12)	C6—C5—C14—C1	179.0 (7)
C5—C6—C7—C8	−178.2 (8)	C4—C5—C14—C13	178.7 (8)
O3—C6—C7—C12	−178.2 (7)	C6—C5—C14—C13	0.7 (11)
C5—C6—C7—C12	3.0 (11)	O4—C13—C14—C1	2.5 (11)
C12—C7—C8—C9	−0.9 (13)	C12—C13—C14—C1	−179.5 (7)
C6—C7—C8—C9	−179.8 (8)	O4—C13—C14—C5	−179.2 (7)
C7—C8—C9—C10	0.1 (14)	C12—C13—C14—C5	−1.1 (11)
C8—C9—C10—C11	0.8 (14)		

### Hydrogen-bond geometry (Å, º)

**Table:**

*D*—H···*A*	*D*—H	H···*A*	*D*···*A*	*D*—H···*A*
O1—H1···O4	0.80 (9)	1.82 (9)	2.536 (8)	148 (9)
O2—H2···O3	0.99 (10)	1.65 (10)	2.568 (9)	152 (8)
C3—H3···O4^i^	0.95	2.46	3.396 (9)	169
C9—H9···O1^ii^	0.95	2.59	3.308 (10)	133
C9—H9···O2^iii^	0.95	2.69	3.394 (10)	132

Symmetry codes: (i) −*x*, −*y*+1, *z*−1/2; (ii) *x*+1/2, −*y*, *z*; (iii) −*x*+1/2, *y*−1, *z*+1/2.
