# Supplementary figures and images for: Crystal structure of 2-bromo-1,4-dihy­droxy-9,10-anthra­quinone
Source: Acta Crystallogr Sect E Struct Rep Online. 2014 Sep 27;70(Pt 10):o1130. doi: 10.1107/S1600536814020996 (PMC4257177; doi:10.1107/S1600536814020996)

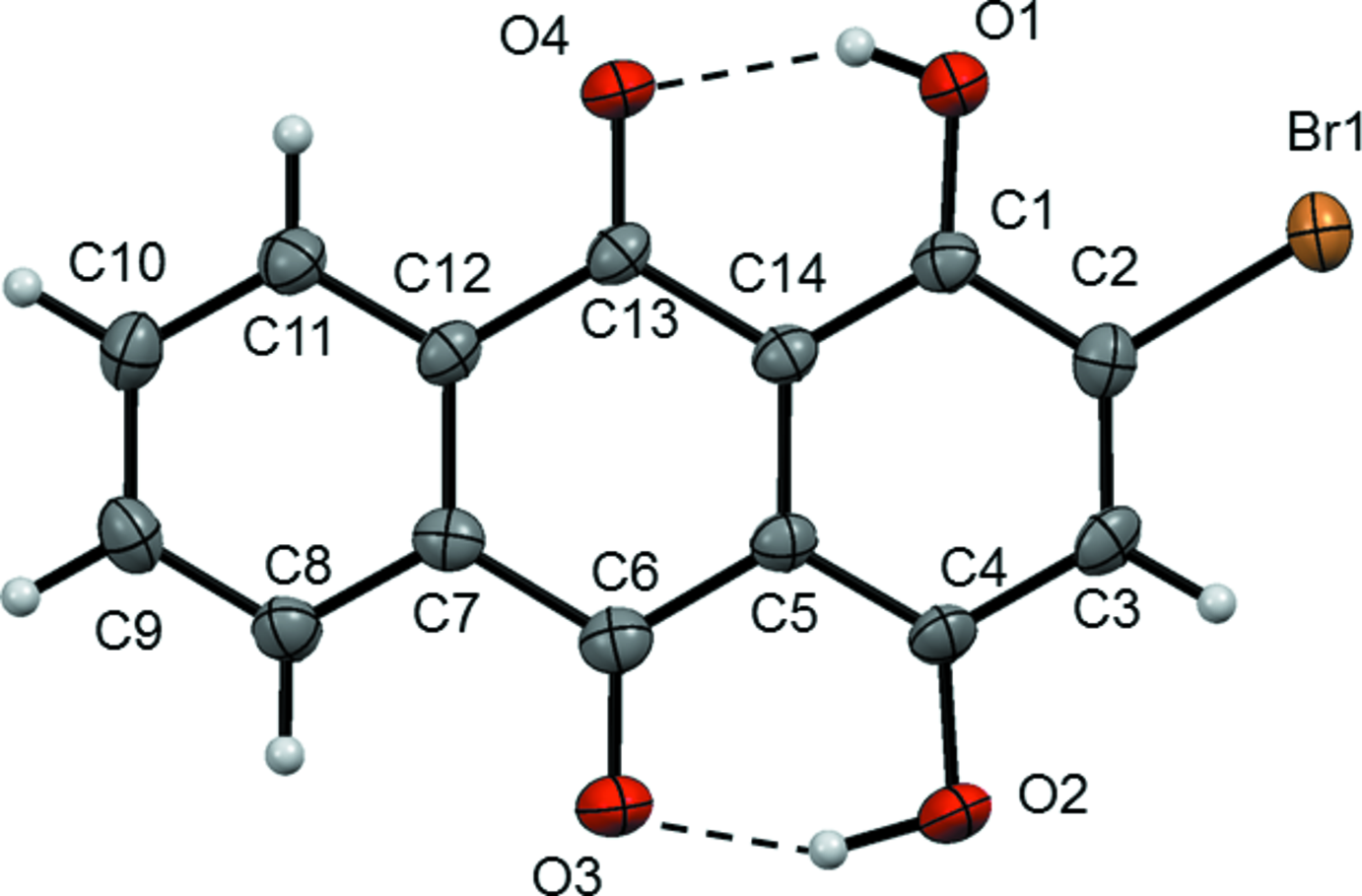

Supplement: Supplementary file 4 [file e-70-o1130-fig1.tif]

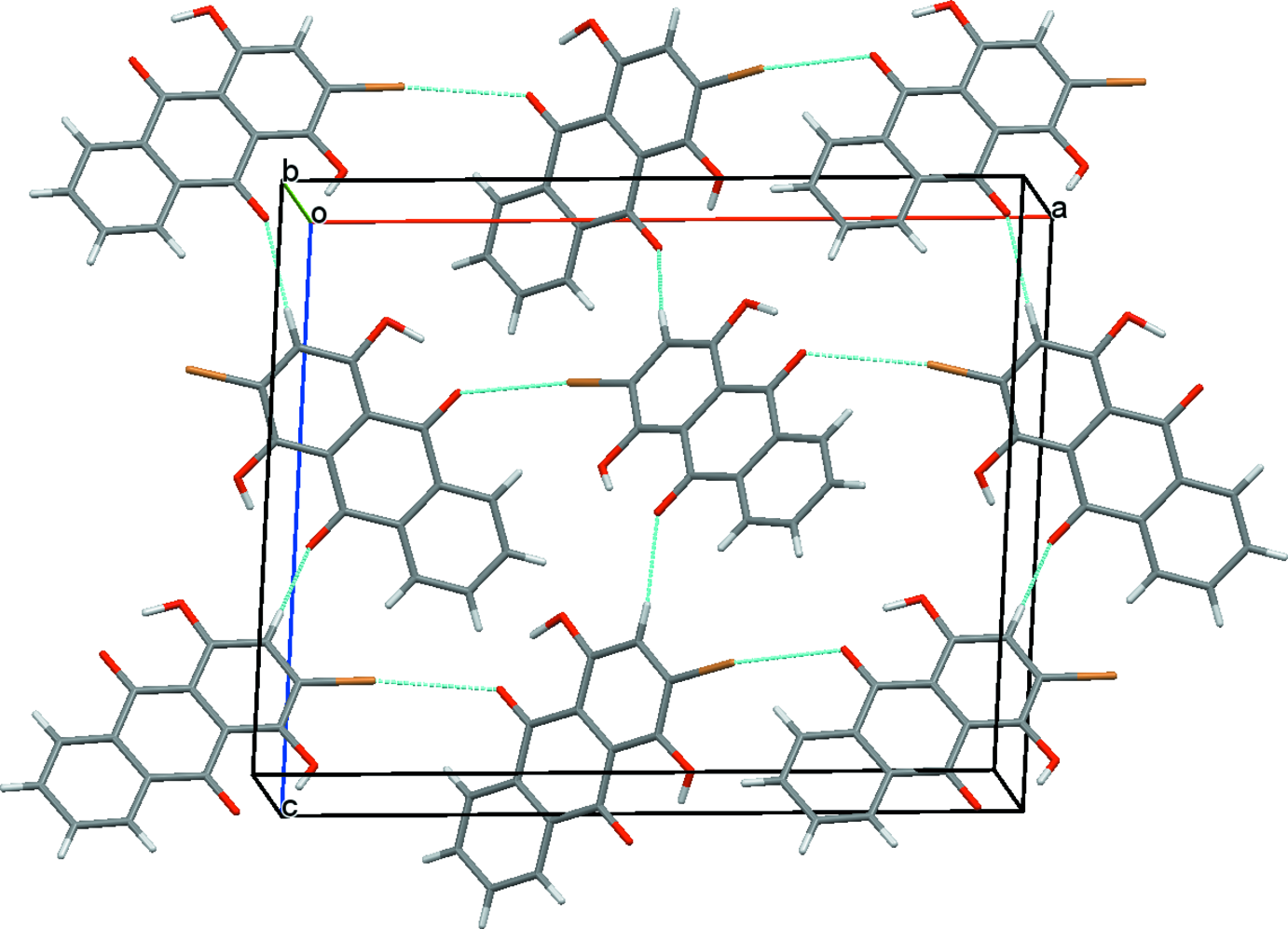

Supplement: Supplementary file 5 [file e-70-o1130-fig2.tif]
